# Brazilian version of the “Primary Sjögren’s Syndrome - Quality of Life questionnaire (PSS-QoL)”: translation, cross-cultural adaptation and validation

**DOI:** 10.1186/s42358-024-00395-7

**Published:** 2024-08-19

**Authors:** Samira Tatiyama Miyamoto, Érica Vieira Serrano, Ana Paula Espíndula Gianórdoli, Lara Betini Altoé, Bianca Domingos Noronha, Pedro Henrique Alves dos Santos, Ana Paula Truhlar Pedrini, Nicole Reis Souza da Silva, Letícia Fonseca Favarato, Luíza Vallory Alochio, Weider Andrade Tomé, Angelika Lackner, Valéria Valim

**Affiliations:** 1 Departamento de Educação Integrada em Saúde, Universidade Federal do Espírito Santo (UFES), Av. Marechal Campos, 1468, Maruípe, Vitória, ES CEP: 29040-090 Brazil; 2 Hospital Universitário Cassiano Antônio Moraes (Hucam), Universidade Federal do Espírito Santo (Ufes), Vitória, ES Brazil; 3 Department of Rheumatology and Immunology, Medical University of Graz, Graz, Austria; 4 Hospital Universitário Cassiano Antônio Moraes (Hucam), Programa de Pós-graduação em Saúde Coletiva (PPGSC), Universidade Federal do Espírito Santo (Ufes), Vitória, ES Brazil

**Keywords:** Sjögren's syndrome, Quality of life, Translation, Validation, Reliability

## Abstract

**Background:**

The Primary Sjögren’s Syndrome Quality of Life questionnaire (PSS-QoL) is the first specific instrument to assess health-related quality of life (HRQoL) in Sjögren’s disease (SjD). The aim is to translate and cross-culturally adapt the PSS-QoL into Brazilian Portuguese and to evaluate its psychometric properties.

**Methods:**

The original English version was translated into Brazilian Portuguese by two native Brazilians who were proficient in the English language. The retranslation was conducted by two native Americans proficient in Brazilian Portuguese. A committee undertook an analysis of the translated and retranslated versions, resulting in the generation of the first Brazilian version, which was submitted to the cross-cultural adaptation phase. In this phase, 50 participants with SjD responded to the instrument in Stages I and II, resulting in the generation of the second and final Brazilian version. To assess the psychometric properties, demographic and clinical data were collected from 75 patients. The HRQoL questionnaires (final Brazilian version of the PSS-QoL, Short Form-36 Health Survey (SF-36) and EuroQoL-5 dimension (EQ-5D)) were completed. Construct validity was analyzed using the Pearson or Spearman correlation coefficient. Reliability was analyzed using Cronbach’s alpha and the intraclass correlation coefficient (ICC).

**Results:**

Eight questions and one response item were revised due to an incomprehension rate of greater than 15% among the participants in the cross-cultural adaptation phase. The final Brazilian version of the PSS-QoL was validated, revealing a high correlation between the total score and functional capacity (*r*= −0.713, *p* < 0.001), and vitality (*r*= −0.770, *p* < 0. 001) and mental health (*r*= −0.742, *p* < 0.001) domains of the SF-36 and a moderate correlation with the other domains of the SF-36 and a moderate correlation with the EQ-5D-tto (*r*= −0.573, *p* < 0.001), and EQ-5D-VAS (*r*= −0.559, *p* < 0.001). The intraobserver (ICC = 0.939; Cronbach’s alpha = 0.964) and interobserver (ICC = 0.965; Cronbach’s alpha = 0.964) reliability of the total score showed very high consistency.

**Conclusion:**

The Brazilian version of the PSS-QoL has been demonstrated to be a valid and reproducible instrument for the assessment of HRQoL in patients with SjD.

**Supplementary Information:**

The online version contains supplementary material available at 10.1186/s42358-024-00395-7.

## Background

Sjögren’s disease (SjD) is a systemic autoimmune disease that is characterized by lymphocytic infiltration and progressive destruction of the exocrine glands. The disease is clinically characterized by the presence of dryness in the mouth and eyes. A considerable proportion of patients present with migrating joint pain and fatigue, while 40% of SjD patients exhibit systemic manifestations [1]. The disease predominantly affects middle-aged women, with a female-to-male ratio of 9:1. The estimated prevalence of primary disease is between 0.04 and 0.17 [2].

The concept of health-related quality of life (HRQoL) is defined in various ways. One definition is as an individual’s functional capacity and their perceived well-being in relation to physical, mental, and social domains. Furthermore, it encompasses the impact of all health-related factors on an individual’s life [3]. The role of HRQoL in medical decision-making is becoming increasingly prominent, with data derived from this concept being employed to assess the relative value of interventions for different diseases. This allows for the economic allocation of health resources. It is a clinical outcome reported by patients (patient-reported outcome, PRO) and is increasingly utilized in clinical trials.

The Miyamoto et al. review from our group demonstrated that the HRQoL of patients with SjD was significantly lower than that of healthy controls or individuals with normative data from the general population in all studies, regardless of the HRQoL tool used. Our findings indicate that impaired HRQoL is associated with a range of factors, including the main symptoms of dryness, pain and fatigue, as well as autonomic response, physical function and activity level, pruritus, and psychological, oral, ocular, sexual and sleep disorders. Studies that have focused on oral and ocular involvement have demonstrated that these factors have a significant impact on HRQoL. Nevertheless, a number of other more general studies that have employed multivariate analysis have demonstrated that dryness is less predictive of HRQoL than pain or fatigue. This may be indicative of the limitations of single visual analogue scales (VAS), which are frequently employed in these studies. A further limitation of the studies reviewed is that some of them included only a small number of patients. In general, a small association was observed between systemic disease activity (as measured by the Sjogren’s Syndrome Disease Activity Index, ESSDAI) and HRQoL. However, pulmonary involvement has been demonstrated to be associated with impaired HRQoL [4].

The two most commonly employed generic measures of HRQoL are the Short-Form Health Survey (SF-36) and the EuroQoL-5 dimension (EQ-5D). Furthermore, the RAND 36-item Health Survey (RAND-36) and the World Health Organization Quality of Life Instrument – Short Form (WHOQOL-BREF) are also employed. These tools offer the advantage of being well understood and allowing comparisons across diseases [4]. Nevertheless, the complex and multifaceted nature of SjD symptomatology suggests the necessity for the utilization of a disease-specific tool, such as the Primary Sjögren’s Syndrome - Quality of Life questionnaire (PSS-QoL) [5], in order to accurately assess the association and impact of HRQoL.

The PSS-QoL, a PRO questionnaire, is the first specific instrument for the assessment of patients’ HRQoL in SjD. The PSS-QoL comprises 25 questions and can be divided into two main categories: physical (discomfort and dryness) and psychosocial. The internal consistency of the PSS-QoL was demonstrated by a Cronbach’s alpha of 0.892. Correlations between the PSS-QoL and the Sjogren’s Syndrome Patient Reported Index (ESSPRI) (*r* = 0.755) and between the PSS-QoL and the Eq. 5D-pain/discomfort (*r* = 0.531) were found to be strong and moderate, respectively. The reliability of the PSS-QoL was demonstrated by an intraclass correlation coefficient (ICC) of 0.958 (95% CI 0.926–0.981) [5].

As observed by Guillemin et al., the extensive number of studies conducted in various languages, predominantly English, necessitates the availability of assessment instruments in the first language of the population being assessed in order to complement the diagnosis. This can be achieved through the development of new instruments or through the translation and adaptation of existing instruments. The latter is the least labor-intensive, costly, and time-consuming option [6].

The aim of this study was to translate and cross-culturally adapt the PSS-QoL into Brazilian Portuguese and evaluate the psychometric properties (reliability and construct validity) of the Brazilian version.

## Methods

### Location and sample

A cross-sectional observational study was conducted at the Sjögren’s disease outpatient clinic of the rheumatology service at a tertiary hospital located in the city of Vitoria, Espirito Santo, Brazil.

In the cross-cultural adaptation phase, five individuals were selected for each domain of the PSS-QoL. A total of 50 participants with a diagnosis of SjD according to the classification criteria of the American-European Group Consensus 2002 and/or American-European Consensus 2016 [7] were randomly selected for Stage I and II [8]. In the psychometric property evaluation phase, the sample consisted of 75 participants.

Participants who had previously been diagnosed with other conditions that manifested as xerophthalmia or xerostomia, such as hepatitis C, acquired immunodeficiency syndrome, past head and neck radiotherapy, graft-versus-host disease, sarcoidosis or hyper IgG4 syndrome; and who had been diagnosed with other connective tissue diseases were excluded from the study.

### Characterization of the sample

The following data were collected: sociodemographic data (age, sex, ethnicity, education level, occupation and marital status) and clinical data (date of first symptoms and diagnosis, disease activity assessed by the ESSDAI [9], symptoms assessed by the ESSPRI [10] and damage assessed by the Sjögren’s Syndrome Disease Damage Index (SSDDI) [11]).

### Translation and cross-cultural adaptation procedures

The methodology employed for the translation and cross-cultural adaptation phase was in accordance with the approach proposed by Guillemin et al. [6].

The original version of the PSS-QoL was translated into Brazilian Portuguese language by two native Brazilians who were proficient in English and were familiar with the purpose of the study. A committee comprising two physiotherapists and two physicians proficient in English and with expertise in the disease in question analyzed the two translations and generated the first Brazilian version. Subsequently, this version was retranslated into English by two native Americans proficient in Brazilian Portuguese who were unaware of the purpose of the study and the original instrument. The two back-translated versions were then subjected to comparison with the original version by the committee, which found no relevant differences. Consequently, the first Brazilian version was subjected to the cross-cultural adaptation phase.

In Stages I and II of the cross-cultural adaptation phase, the questionnaire was administered by two trained physiotherapists. As the PSS-QoL is a PRO questionnaire, participants were permitted to respond to the questionnaire independently (self-administration) or request assistance from one of the physiotherapists in reading the questionnaire if they found it challenging [12].

The first Brazilian version was administered to 25 participants in Stage I of the cross-cultural adaptation phase. The alternative “Não entendi” was incorporated into all the questions and response items. All participants were informed that they were prohibited from asking questions during the time being counted. Additionally, participants were instructed to indicate the option “Não entendi” if they were unable to answer a question. Subsequently, the physiotherapists provided clarification, and the participants were able to respond to the remaining questions. Upon identifying that more than 15% of the participants had selected this alternative, the question or answer option was revised by the committee. Only the revised questions/items from the second Brazilian version were applied to a further 25 participants in Stage II. At this stage, the committee may implement further modifications if there is a discrepancy of 15% or more in understanding in any other question or response item. In such an instance, the revised questions or items would have to be reapplied to a further 25 patients.

### Procedures for assessing psychometric properties

The reliability of the final Brazilian version of the PSS-QoL was evaluated through three applications of the instrument. The initial two applications were conducted on the same day by two distinct assessors (assessor 1 and assessor 2) to assess interobserver reliability, with a 20-minute interval between applications, resulting in a total of approximately one hour. The third application was re-evaluated by assessor 1 seven days later at the same time to assess intraobserver reliability.

To assess the construct validity of the Brazilian version of the PSS-QoL, generic HRQoL instruments that have already been validated were applied together, namely the SF-36 [13] and the EQ-5D [14, 15]. This item is analyzed in order to ascertain the degree of certainty regarding the measurement of the instrument in question.

During this phase, the assessors read all the questionnaires in order to guarantee the homogeneity of the methodology. This is necessary since reliability is contingent upon the assessors administering the questionnaires at different times.

### PSS-QoL calculation [5]

The questions pertain to the patient’s experiences within the previous four-week period. The physical dimension comprises a numerical rating scale (ranging from 0 to 10) for discomfort and checkboxes for each physical symptom considered to impair HRQoL.

In order to ascertain the responses to symptom-related questions, it is necessary to utilize a system of checkboxes, with the response of “yes” indicated by a tick. Each affirmative response is assigned a score of one point. The question pertaining to vaginal dryness is intended for responses from women only.

The psychosocial dimension is scored on a 5-point Likert scale, with 14 questions/statements. The possible answers and scores are as follows: never (nunca) = 0, rarely (raramente) = 1, sometimes (às vezes) = 2, often (frequentemente) = 3 and always (sempre) = 4.

The domain scores are as follows: physical—sum of questions 1 to 11 (score 0 to 40); discomfort—sum of question 1 to 6 (score 0 to 15); dryness—sum of questions 7 to 11 (score 0 to 25 for women and 0 to 21 for men); and psychosocial—sum of questions 12 to 25 (score 0 to 56).

The total score of the PSS-QoL ranges from 0 to 96 (for women) and from 0 to 92 (for men, excluding vaginal dryness). There is no established cutoff score for the questionnaire; however, it is generally accepted that higher scores indicate worse HRQoL.

### Statistical analysis

A descriptive analysis was conducted in order to identify the clinical and demographic characteristics of the study population and quantify the prevalence of non-understanding of the questions.

The interobserver and intraobserver reliability of the Brazilian version was analyzed using Cronbach’s alpha and the intraclass correlation coefficient (ICC). The construct validity of the PSS-QoL was analyzed through convergent validity with the SF-36 and EQ-5D. This was accomplished through the use of the Pearson or Spearman correlation coefficient, following the determination of the normality of the variables through the Kolmogorov-Smirnov test. The correlation intensity was rated as negligible (0.30), low (0.30 to 0.50), moderate (0.51 to 0.70), high (0.71 to 0.90), and very high (> 0.90) [16].

## Results

### Translation

The first Brazilian version resulting from the translation, retranslation, and analysis conducted by the committee was subsequently subjected to the cross-cultural adaptation phase.

### Cross-cultural adaptation

Of the 55 participants selected for the cross-cultural adaptation phase, five were excluded from the study because they met one or more of the exclusion criteria.

The first Brazilian version was structured and applied in Stage I of the cross-cultural adaptation phase. The 25 participants in this Stage were primarily female (88%) with an average age of 57 (±12.3) years. In terms of educational level, 40% of the participants had completed high school, 16% had completed middle school, and 12% had completed elementary school (Table 1). The mean PSS-QoL score for women was 45 (±17), with a range of 6 to 75 points. The mean score for men was 31 (±28), with a range of 14 to 68 points. A total of 86% of the selected participants completed the questionnaire independently. The mean response time was 7 min and 8 s (±4.0).

**Table 1:** Sociodemographic and clinical characteristics of the participants in the cross-cultural adaptation and psychometric property phases

		Cross-cultural adaptation		Psychometric properties
		Stage I **(** ***n*** ** = 25)**	Stage II **(** ***n*** ** = 25)**	**(** ***n*** ** = 75)**
Sex, n (%)	Female	22 (88)	25 (100)	71 (95)
	Male	3 (3)	0	4 (5)
Age (years), mean ± SD		57 ± 12.3	51 ± 15.8	53.5 ± 11.1
Ethnicity, n (%)	Mixed	13(52)	15 (60)	43 (57)
	Caucasian	11 (44)	6 (24)	25 (33)
	Asian	1 (4)	0	0
	African	0	3 (12)	7 (9)
	Indigenous	0	1 (4)	0
Education level, n (%)	Elementary school	3 (12)	2 (8)	
	Incomplete middle school	3 (12)	5 (20)	
	Middle school	4 (16)	2 (8)	
	Incomplete high school	0	1 (4)	
	High school	10 (40)	8 (32)	
	Higher education	4 (16)	5 (20)	
	Illiterate	1 (4)	1 (4)	
Occupation status, n (%)	Employed	8 (32)	7 (28)	38 (51)
	Unemployed	2 (8)	7 (28)	1 (1)
	Retired	11 (44)	4 (16)	25 (33)
	Household	4 (16)	7 (28)	10 (13)
Marital status, n (%)	Single	4 (14)	7 (28)	42 (56)
	Married	14 (56)	9 (36)	13 (17)
	Divorced	3 (12)	5 (20)	19 (25)
	Widowed	4 (16)	3 (12)	0
Time of symptoms (years), mean ± SD		9 ± 9	9.5 ± 5.2	11.7 ± 7.0
Time of disease (years), mean ± SD		6 ± 6	4 ± 4.9	8.4 ± 5.6

*n* sample, *SD* standard deviation

In response to the question, “3. Eu tive dores migratórias recorrentes no meu corpo,” eight (32%) of the participants demonstrated a lack of comprehension regarding the meaning of “dores migratórias recorrentes.”

For question “4. Constipação”, five (20%) of the participants indicated that they lacked an understanding of the term or confused it with nasal constipation.

In question 10 regarding the nose, four (16%) participants demonstrated a lack of comprehension regarding the meaning of the item “Mudança no olfato”.

In response to the question, “12. Sou a única pessoa com essas queixas,” three (12%) participants indicated uncertainty regarding the appropriate response, one (4%) lacked familiarity with the term “queixa,” and one (4%) inquired as to whether the physiotherapists wished to know if she had discussed her concerns with others.

With regard to the question “17.”, In answer to the question “Sou retraído?”, five (20%) participants stated that they did not understand the meaning of the word “retraído.”

In response to the question “18. Estou preocupado (a) com efeitos colaterais”, a total of five participants (20%) indicated that they were unaware of the meaning of “efeitos colaterais”.

After Stage I, five questions (questions 3, 4, 12, 17 and 18) and one response item (item 10A) were identified as being incomprehensible by more than 15% of the participants. Consequently, the committee initiated a process of reformulation with the objective of generating the second Brazilian version. Only the revised questions/items from the second Brazilian version were applied to a further 25 participants in Stage II of the cross-cultural adaptation phase. This process was conducted with 100% female participants, with an average age of 51 years (ranging from 35 to 75 years) and with a similar level of education to that of the participants in Stage I (Table 1). In light of the fact that the initial wording of questions 12, 13, 14, and 15 was the same [5], the committee decided to modify three additional questions (questions 13, 14, and 15) despite the percentage being below 15%. This was implemented in order to ensure uniformity in the introductory wording of these questions, as had been done in the case of question 12, which had a misunderstanding rate of 20%.

In Stage II, all the questions demonstrated a percentage of non-understanding that was less than 15% (see Additional file 1). Overall, 18 (72%) participants exhibited some difficulty in responding to the questionnaire in Stage I. In Stage II, this number decreased to nine (36%) participants. The second Brazilian version was considered the final Brazilian version.

### Psychometric properties

Of the 93 participants selected for the psychometric property evaluation phase, 11 were excluded due to their fulfillment of some of the exclusion criteria, and 7 were excluded due to their failure to undergo re-evaluation. A total of 75 participants were included in the study. The majority of participants were women (92%), with an average age of 53.5 years (± 11.1). The majority were married (48%) and had an average diagnosis time of 8.4 years (± 5.6) (Table 1). On average, the participants exhibited low disease activity, as indicated by the ESSDAI (2.36 ± 3.5), and symptoms that were considered mildly unacceptable on the ESSPRI (4.91 ± 3.9). With regard to the SF-36 HRQoL domains, the lowest scores were observed for role-physical (42.2 ± 42.9), general health (43.3 ± 22.7) and role-emotional (44.8 ± 45.5) (Table 2).

**Table 2:** Clinical characteristics and health-related quality of life of patients with Sjögren’s disease in the psychometric property evaluation phase (*n* = 75)

Variables	*n* (%)	Mean ± SD
ESSDAI		2.36 ± 3.6
SSDDI		1.95 ± 1.8
ESSPRI Total		4.91 ± 3.9
ESSPRI fatigue		4.81 ± 3.4
ESSPRI pain		4.01 ± 3.5
ESSPRI dryness		4.78 ± 3.5
Schirmer test, Positive	48 (64)	
Focal lymphocytic sialodenitis, Positive	56 (74.7)	
Unstimulated Whole Salivary Flow, Positive	56 (74.7)	
Anti-Ro, Positive	50 (66.7)	
Anti-La, Positive	21 (23)	
SF-36 Physical functioning		57.4 ± 29.4
SF-36 Role-physical		42.2 ± 42.9
SF-36 Bodily-pain		47.9 ± 28.6
SF-36 General health		43.3 ± 22.7
SF-36 Vitality		47.9 ± 26.1
SF-36 Social functioning		60.0 ± 29.6
SF-36 Role-emotional		44.8 ± 45.5
SF-36 Mental health		55.9 ± 26.3
EQ-5D - tto		0.48 ± 0.4
EQ- 5D - VAS		65.2 ± 21.9

*n* sample, *SD* standard deviation, *ESSDAI* EULAR Sjögren’s syndrome disease activity index, *SSDDI* Sjögren’s Syndrome Disease Damage Index, *ESSPRI* EULAR Sjögren’s syndrome patient reported index, *EQ-5D - tto* EuroQoL-5 dimension - time trade-off, *EQ-5D - VAS* EuroQoL-5 dimension - visual analog scale, *SF-36* Short Form-36 Health Survey

The patients required approximately 6.57 min (±2.25) to complete the final Brazilian version with the assessor 1, 7.13 min (±2.56) with the assessor 2 and 5.93 min (±2.56) at the reassessment. The mean total and domain scores are presented in Table 3.

**Table 3:** Mean scores of the domains and total score of the final Brazilian version of the Primary Sjögren’s Syndrome Quality of Life questionnaire (PSS-QoL)

Domains (range)	Assessor 1	Assessor 2	Reassessment
	Mean ± SD	Mean ± SD	Mean ± SD
Physical (0–40)	18.57 ± 8.76	17.50 ± 8.82	18.34 ± 8.42
Discomfort (0–15)	8.06 ± 4.15	7.72 ± 4.06	7.78 ± 3.82
Dryness (0–25*; 0–21**)	10.50 ± 5.53	9.11 ± 5.36	9.77 ± 5.04
Psychosocial (0–56)	27.18 ± 11.55	28.03 ± 11.08	27.85 ± 10.77
Total (0–96*; 0–92**)	45.75 ± 18.60	45.68 ± 18.18	46.19 ± 17.48
Time (minutes)	6.57 ± 2.25	7.13 ± 2.56	5.93 ± 2.56

*SD* standard deviation

*range score for women

**range score for men

The intraobserver reliability values of the final Brazilian version of the PSS-QoL demonstrated a high correlation in the domains of discomfort and dryness. For the remaining items, the correlation was very high. Interobserver reliability exhibited very high agreement for all items (Table 4).

**Table 4:** Reliability of the domains and total score of the final Brazilian version of the Primary Sjögren’s Syndrome Quality of Life questionnaire (PSS-QoL)

	ICC		Cronbach’s alpha	
	Intraobserver	Interobserver	Intraobserver	Interobserver
Physical	0.912	0.973	0.915	0.977
Discomfort	0.863	0.961	0.866	0.963
Dryness	0.871	0.951	0.882	0.966
Psychosocial	0.914	0.938	0.913	0.938
Total	0.939	0.965	0.939	0.964

The construct validation of the final Brazilian version of the PSS-QoL revealed a high correlation with the physical functioning, vitality and mental health domains of the SF-36 and a moderate correlation with the other domains. With the exception of the low correlation between the discomfort, dryness and physical domains of the PSS-QoL and the role-emotional domain of the SF-36, and between the dryness domain of the PSS-QoL and the physical functioning domain of the SF-36 and VAS of the EQ-5D, all the other correlations were high or moderate (Table 5).

**Table 5:** Correlations between the final Brazilian version of the Primary Sjögren’s Syndrome Quality of Life questionnaire (PSS-QoL) and the short Form-36 Health Survey (SF-36) and EuroQoL-5 dimension (EQ-5D) questionnaires

	Discomfort	Dryness	Physical	Psychosocial	PSS-QoL total
SF-36 Physical functioning	−0.443**	−0.535**	−0.582**	−0.687**	−0.713**
SF-36 Role-physical	−0.445**	−0.350**	−0.468**	−0.562**	−0.574**
SF-36 Bodily-pain	−0.680**	−0.496**	−0.671**	−0.538**	−0.656**
SF-36 General health	−0.529**	−0.433**	−0.545**	−0.614**	−0.644**
SF-36 Vitality	−0.579**	−0.507**	−0.617**	−0.762**	−0.770**
SF-36 Social functioning	−0.634**	−0.417**	−0.580**	−0.679**	−0.688**
SF-36 Role-emotional	−0.359**	−0.335**	−0.374**	−0.614**	−0.566**
SF-36 Mental health	−0.538**	−0.426**	−0.538**	−0.777**	−0.742**
EQ-5D - tto	−0.493**	−0.412**	−0.548**	−0.522**	−0.573**
EQ- 5D - VAS	−0.514**	−0.344**	−0.472**	−0.548**	−0.559**

*EQ-5D - tto* EuroQoL-5 dimension - time trade-off, *EQ-5D - VAS* EuroQoL-5 dimension - visual analog scale

**p < 0.001

## Discussion

This study translated and cross-culturally adapted the Primary Sjögren’s Syndrome Quality of Life questionnaire (PSS-QoL) into Brazilian Portuguese and assessed the psychometric properties of the Brazilian version (see Additional file 2), including reliability and construct validity.

The characteristics of the sample in this study were comparable to those of the study by Lackner et al. [5], which developed the PSS-QoL. In that study, the majority of participants (90.7%) were women, with an average age of 58.5 (±12.5) years. The average ESSDAI was 2.0 (±2.5), the average ESSPRI was 4.3 (±2.0), and the average disease duration was 4.8 (±4.1) years.

In Stage I of the cross-cultural adaptation phase, the PSS-QoL score ranged from 6 to 75 points, which is consistent with the findings of the study by Lackner et al. [5], which reported scores between 3 and 76 points. These values indicate that the health condition and its clinical manifestations have a mild to intermediate impact on patients’ HRQoL.

In the Brazilian version, participants took approximately seven minutes to complete the PSS-QoL questionnaire in Stage I of the cross-cultural adaptation phase and in the psychometric property phase, which was two minutes longer than the participants in the study by Lackner et al. [5], who took approximately five minutes. The longer completion time observed in this study may be attributed to the fact that 40% of the participants reported having completed only elementary and middle school. This percentage may be attributed to the study’s setting in a public hospital, where 4% of the sample lacked literacy, which aligns with the national average of 7% observed in the general Brazilian population [17]. The Lackner et al. study [5] did not provide information regarding the educational level of its participants. However, the study only included individuals with sufficient German language skills.

At the outset of the study, the title of the PSS-QoL in the original questionnaire “Quality of life questionnaire for patients with primary Sjögren’s syndrome” was translated to “Questionário de qualidade de vida para pacientes com síndrome de Sjögren primária”. However, due to the new nomenclature of the disease (Sjögren’s disease) [18], the title of the Brazilian version became “Questionário de qualidade de vida para pacientes com doença de Sjögren”.

Some of the terms utilized in the query, “3 Eu tive dores migratórias recorrentes no meu corpo,” are not frequently encountered in the everyday lives of a substantial portion of Brazil’s population. Consequently, the terms were modified to enhance comprehension. To the words “migratórias recorrentes,” the description “dor que aparece e desaparece em diferentes partes do corpo” was added, which resulted in a reduction in the number of individuals who were unable to comprehend the question from 32% in Stage I to 4% in Stage II of the cross-cultural adaptation phase.

A significant number of participants interpreted the term “constipação” as denoting nasal rather than intestinal constipation. Consequently, incorporating the term “intestinal” into the definition resulted in a notable decrease in uncertainty, from an initial 20% in Stage I to 12% in Stage II of the cross-cultural adaptation phase.

In response to the item titled “10. Mudança no olfato,” the term “olfato” is not utilized colloquially by the general public. As an illustration, following the incorporation of the phrase “dificuldade de sentir o cheiro,” the proportion of uncertainties declined from 16% in Stage I to 4% in Stage II of the cross-cultural adaptation phase.

It is important to note that questions 12 to 15 were presented collectively in a single cell of the Table [5]. The expression “Eu tenho sentido que.” was present only at the top of the table row. Consequently, a considerable number of patients failed to observe the correct positioning of this element during the Stage I of the cross-cultural adaptation phase. Following the reorganization, these questions were presented in separate rows. Furthermore, the expression “Eu tenho sentido que.” was integrated at the outset of each question in Stage II. This modification resulted in a notable reduction in the proportion of individuals who expressed uncertainty in responding to question 12, from 20 to 4%. However, the percentage of incomprehension remained below 15% in both stages for questions 13 to 15.

In response to the question “17. Sou retraído” the term “retraído” is also not commonly used. However, after the implementation of “fechado ou isolado” the percentage decreased from 20% in Stage I to 0% in Stage II of the cross-cultural adaptation phase.

The expression “dos medicamentos” was incorporated into the question “18. Estou preocupado (a) com efeitos colaterais dos medicamentos” resulted in a reduction in the percentage of doubts from 20% in Stage I to 0% in Stage II of the cross-cultural adaptation phase. This modification made it evident that the adverse effects described were attributable to the medication and not to the underlying disease, which was a pertinent inquiry for the participants.

As in the study by Lackner et al. [5], the validity was considered high (functional capacity (*r*=−0.713, *p* < 0.001), vitality (*r*=−0.770, *p* < 0. 001) and mental health (*r*=−0.742, *p* < 0.001) domains of the SF-36) to moderate (other domains of the SF-36, EQ-5D-tto (*r*=−0.573, *p* < 0.001), and EQ-5D-VAS (*r*=−0.559, *p* < 0.001)) and the consistency of reliability was very high (intraobserver: ICC = 0.939; Cronbach’s alpha = 0.964; interobserver: ICC = 0.965; Cronbach’s alpha = 0.964) in the present study.

In general, there is a small correlation between systemic disease activity (as assessed by ESSDAI) and HRQoL [4]. Even when the average disease activity is low, HRQoL can be negatively influenced [19–21]. Studies have demonstrated that xerophthalmia has a greater impact on HRQoL than other common symptoms, with a notable effect on emotional, physical, and financial well-being [22]. Nevertheless, mental health does not appear to be influenced by xerophthalmia symptoms, regardless of the severity of the disease [23]. A poorer oral health-related quality of life (OHRQoL) is associated with dysphagia, difficulty speaking, dental caries [24], dysgeusia, burning sensation in the tongue, halitosis [25], taste threshold [26], and oral lesions [24, 27]. The emotional aspect is the SF-36 domain most strongly associated with oral discomfort [28], and it also affects patients’ financial well-being [24]. Chronic itching in the anterior region of the legs, back, and forearms was found to interfere with the emotional domain, with the worst scores being “aggravated by temperature or seasonal changes” and “need to scratch” [29]. Patients with vaginal dryness exhibit a diminished quality of sexual life, with the domains of pain, lubrication, desire, and arousal [30] being most affected. They experience greater sexual distress and engage in less frequent sexual activity than controls [31, 32]. Furthermore, vaginal dryness has a significant impact on the mental and social [33] components of HRQoL [34].

Studies have shown that the greater the fatigue is, the worse the HRQoL in patients with SjD [35]. This is evidenced by lower scores for both the physical [36, 37] and mental components of HRQoL [37]. Moreover, pain is also associated with lower scores on the physical component [36, 37]. A Brazilian study demonstrated that the primary predictors of poor HRQoL in patients with SjD were pain and fatigue, regardless of disease activity, age, education, work disability, marital status, and fibromyalgia [20]. Over time, it has been observed that fatigue remains stable, whereas there is an age-related decline in HRQoL [38]. However, one study observed an improvement in vitality that may have occurred due to patients acquiring effective coping strategies [39]. Higher levels of anxiety and depression in SjD patients have been found to be associated with lower HRQoL [40].

The lengthy process of diagnosis, the quality of interaction with healthcare professionals, the complex and varied manifestations of the disease, and the ability to cope positively with chronic illness [41] are all factors that affect the HRQoL of patients with SjD [42]. Additionally, difficulties at work, limited eating, and restrictions in social life all contribute to this effect [42]. A study of SjD patients from five European countries revealed a range of limitations in daily life, including self-care, productivity, and leisure activities [43].

Regardless of the assessment tool utilized, HRQoL is significantly diminished in SjD patients in numerous studies conducted across diverse countries when compared to the HRQoL of healthy controls [4]. This reduction in HRQoL is comparable to that observed in other chronic diseases, including rheumatoid arthritis [44, 45], systemic lupus erythematosus [36], fibromyalgia [44], and, intriguingly, non-Sjögren’s disease sicca syndrome [36, 46].

Lackner et al. [21] demonstrated a significant negative correlation between PSS-QoL-dryness and Schirmer’s test (*r* = − 0.31, *p* < 0.05) and the stimulated salivary flow test (*r* = − 0.390, *p* < 0.01), but not between the ESSPRI-dryness and any objective dryness test. This suggests that an overall VAS such as the ESSPRI might not allow a precise evaluation of the patient perspective of dryness. In addition, two distinct groups of SjD patients were identified: (1) those with high perceived dryness and impaired HRQoL, and (2) those with a lower perception of dryness but higher clinical and immunological disease activity. The association between lower perceived dryness and higher immunological activity, as determined by increased levels of IgG, free light chains κ and λ, and rheumatoid factor IgA, was observed.

The PSS-QoL offers a number of advantages. Firstly, it enables the assessment of the dryness of all affected body parts separately, as well as the additional symptoms related to dryness. Secondly, it allows for the assessment of other important HRQoL domains that affect the daily lives of patients with SjD. These include physical discomfort (pain, fatigue and digestive), as well as psychosocial aspects. In this study, the PSS-QoL was compromised by at least 50% across all domains. Lendrem et al. demonstrated a statistically significant relationship between EQ-5D utility values and pain and depression scores in patients with SjD. Nevertheless, the relative contribution of fatigue and dryness symptoms to overall EQ-5D utility values was minimal. The authors suggest that this may reflect the inherent insensitivity of generic HRQoL instruments in capturing the impact of such symptoms on HRQoL [19]. Although studies on SjD have typically employed generic measures such as the SF-36 and the EQ-5D, the complex and multifaceted nature of SjD symptomatology underscores the necessity for a disease-specific instrument to accurately assess the association and impact of SjD on HRQoL [4]. Consequently, it is plausible that the HRQoL of SjD patients, as measured by generic instruments in previous studies, may be underestimated.

In clinical practice, PRO questionnaires, such as the PSS-QoL, can be self-administered or administered by an interviewer when the participant has a disability or difficulty [12]. Nevertheless, in scientific research, the measurement method must be standardized and carefully chosen, depending on the target population, the aim of the investigation, and the cognitive and clinical conditions of the participants.

It should be noted that this study is not without its limitations. The sample size is relatively small, and the participants were recruited from a single center, which may limit the generalizability of the findings. The mean disease activity of the sample was low, and it was not possible to perform a subgroup stratification analysis. Although all questionnaires were read to participants during the psychometric properties phase, the level of education was not assessed, which may have affected comprehension.

## Conclusion

The “Primary Sjögren’s Syndrome Quality of Life questionnaire (PSS-QoL)” was translated and cross-culturally adapted into Brazilian Portuguese and named the “Questionário de qualidade de vida na doença de Sjögren.” In addition to its status as a specific instrument, the PSS-QoL is also quick to respond, easy to calculate, and does not require a register to be used. The PSS-QoL is a valid and reproducible HRQoL questionnaire for the Brazilian Portuguese language.

## Electronic supplementary material

Below is the link to the electronic supplementary material.

Supplementary Material 1

Supplementary Material 2

## Data Availability

The datasets used and/or analyzed during the current study are available from the corresponding author upon reasonable request.

## Abbreviations

EQ-5D: EuroQoL-5 dimension
ESSDAI: Sjogren’s Syndrome Disease Activity Index
ESSPRI: Sjogren’s Syndrome Patient Reported Index
HRQoL: Health-related quality of life
PRO: Patient-Reported Outcome
PSS-QoL: Primary Sjögren’s Syndrome - Quality of Life questionnaire
RAND-36: RAND 36-item Health Survey
SF-36: Short-Form Health Survey
SjD: Sjögren’s disease
SSDDI: Sjögren’s Syndrome Disease Damage Index
VAS: Visual analog scales
WHOQOL-BREF: World Health Organization Quality of Life Instrument - Short-Form
