# Supplementary material for: Brazilian version of the “Primary Sjögren’s Syndrome - Quality of Life questionnaire (PSS-QoL)”: translation, cross-cultural adaptation and validation
Source: Adv Rheumatol. 2024 Aug 19;64:60. doi: 10.1186/s42358-024-00395-7 (PMC13488849; doi:10.1186/s42358-024-00395-7)
Supplement: Supplementary file 1 [file 42358_2024_395_MOESM1_ESM.pdf]

Additional file 1. Questions and response items of the original version and percentage of nonunderstanding of the questions and response items of the Brazilian version of stages I and II in the cross-cultural adaptation phase of the Primary Sjögren's Syndrome Quality of Life questionnaire (PSS-QoL).

**Original version**	**Translated version (Stage I)**	**No (%)**	**Translated version (Stage II)**	**No (%)**
Quality of life questionnaire for patients with primary Sjögren’s syndrome	Questionário de qualidade de vida para pacientes com síndrome de Sjögren primária	0	-	-
These questions pertain to your complaints within the last four weeks:	Estas perguntas são relacionadas às suas queixas nas últimas 4 semanas:	0	-	-
1.How severe is your pain	1.Qual foi a intensidade da sua dor?	3 (12)	-	-
2.I had joint pain	2.Eu tive dor nas juntas:	0	-	-
3.I had recurrent wandering pains.	3.Eu tive dores migratórias recorrentes no meu corpo:	8 (32)	3.Eu tive dores migratórias recorrentes no meu corpo: (dores que aparecem e desaparecem em diferentes partes do corpo)	1 (4)
I had digestive problems.	Eu tive problemas digestivos	0	-	-
4.Constipation:	4.Constipação:	5 (20)	4.Constipação Intestinal	3 (12)
5.Stomach / abdominal pain:	5.Dor abdominal/estomacal:	0	-	-
6.I had sleeping problems:	6.Eu tive problemas no sono	0	-	-
7.Does your mouth feel dry?	7.Você tem sentido sua boca seca?	0	-	-
If yes: Which of these complaints did you have? (Check all that apply)	Se sim: Você teve alguma dessas queixas? (Marque todas que se aplicam)	0	-	-
( ) Burning sensation in the mouth	( ) Sensação de queimação na boca	2 (8)	-	-
( ) Trouble speaking	( ) Dificuldade para falar	0	-	-

( ) Dental problems	( ) Problemas dentários	0	-	-
( ) Changes in sense of taste	( ) Alterações no paladar	0	-	-
( ) Difficulty eating dry foods	( ) Dificuldade em comer alimentos secos	0	-	-
( ) Compulsion to drink during the night	( ) Necessidade de beber líquidos durante a noite	0	-	-
8.Do your eyes feel dry?	8.Você tem sentido seus olhos secos?	0	-	-
If yes: Which of these complaints did you have? (Check all that apply)	Se sim: Você teve alguma dessas queixas? (Marque todas que se aplicam	0	-	-
( ) Recurring inflammation	( ) Inflamações frequentes (olhos avermelhados)	0	-	-
( ) Pain	( ) Dor	0	-	-
( ) Gritty Feeling	( ) Sensação de areia	0	-	-
( ) Crusty eyes / eyelids	( ) Excesso de secreção (remela)	0	-	-
( ) Deterioration of vision	( ) Piora da visão	4	-	-
( ) No tears (inability to cry)	( ) Piora da visão	0	-	-
( ) Everyday activities like driving, reading and watching TV are limited or impossible.	( ) As atividades diárias como dirigir, ler ou assistir televisão estão limitadas ou impossibilitadas	1 (4)	-	-
9.Does your skin feel dry?	9.Você tem sentido a sua pele seca?	2 (8)	-	-
If yes: Did you have either of these complaints? (Check what apply)	Se sim: Você teve alguma dessas queixas? (Marque todas que se aplicam)	0	-	-
( ) Skin reddening	( ) Vermelhidão da pele	1 (4)		
( ) Tight, taut skin	( ) Pele esticada	2 (8)	-	-
10.Does your nose feel dry?	10.Você tem sentido o seu nariz seco?	0	-	-
If yes: Did you have either of these complaints? (Check what apply)	Se sim: Você teve alguma dessas queixas? (Marque todas que se aplicam)	0	-	-

( ) Change in sense of smell	( ) Mudança no olfato	4 (16)	Mudança no olfato (dificuldade de sentir o cheiro)	1 (4)
( ) Nose bleed	( ) Sangramento nasal	0	-	-
The following question is for women only:	A próxima pergunta é somente para as mulheres	0	-	-
11.Does your vagina feel dry?	11.Você tem sentido a sua vagina seca?	0	-	-
If yes: Did you have any of these complaints? (Check all that apply)	Se sim: Você teve alguma dessas queixas? (Marque todas que se aplicam)	0	-	-
( ) Any kind of vaginal pain	( ) Algum tipo de dor vaginal	0	-	-
( ) Itching	( ) Coceira	0	-	-
( ) Pain with intercourse	( ) Dor na relação sexual	0	-	-
Please check to indicate how these statements apply to you:	Indique abaixo quais dessas situações se aplicam a você:	0	-	-
I have a feeling that	Eu tenho sentido que	0	-	-
12.I am the only person with these complaints	12.Sou a única pessoa com essas queixas	5 (20)	12.Eu tenho sentido que sou a única pessoa com essas queixas.	1 (4)
13.My complaints are not taken seriously	13.Minhas queixas não são levadas a sério	2 (8)	13.Eu tenho sentido que minhas queixas não são levadas a sério	2 (8)
14.My complaints are too much for me.	14.Minhas queixas são demais pra mim	0	14.Eu tenho sentido que minhas queixas são demais pra mim	0
15.My family and friends are understanding	15.Minha família e meus amigos me entendem	0	15.Eu tenho sentido que minha família e meus amigos me entendem.	0
16. I am too tired to fulfill obligations to my family and friends	16. Estou cansado demais para cumprir obrigações com minha família e amigos	1 (4)	-	-
17. I am withdrawn	17. Sou retraído	5 (20)	17.Sou retraído, fechado ou isolado.	0
18.I am concerned about side effects	18.Estou preocupado (a) com efeitos colaterais	5 (20)	18.Estou preocupado (a) com efeitos	0

			colaterais dos medicamentos.	
19.I worry about the further course of my disease	19.Preocupo-me com a progressão da minha doença	1 (4)	-	-
20.I have a good feeling about my body	20.Sinto-me bem com o meu corpo	0	-	-
21. I cannot manage my everyday life as well as I did before I became ill	21. Não consigo lidar com a minha vida diária tão bem como fazia antes de adoecer	1 (4)	-	-
22.I tire easily	22.Eu me canso facilmente	0	-	-
23.Everyday activities like driving, work, household and sports are a challenge	23.Atividades rotineiras como dirigir, trabalhar, tarefas domésticas e esportes são um desafio	1 (4)	-	-
24.Remedies like eye drops, creams and physiotherapy impose a financial burden	24.Tratamentos como colírios, cremes e fisioterapia são uma sobrecarga financeira	4 (16)	-	-
25.The disease has reduced my quality of life	25.A doença tem reduzindo minha qualidade de vida	1 (4)	-	-
