# Supplementary material for: Brazilian version of the “Primary Sjögren’s Syndrome - Quality of Life questionnaire (PSS-QoL)”: translation, cross-cultural adaptation and validation
Source: Adv Rheumatol. 2024 Aug 19;64:60. doi: 10.1186/s42358-024-00395-7 (PMC13488849; doi:10.1186/s42358-024-00395-7)
Supplement: Supplementary file 2 [file 42358_2024_395_MOESM2_ESM.pdf]

Additional file 2. Brazilian version of the Primary Sjögren's Syndrome Quality of Life Questionnaire (PSS-QoL) - Questionário de qualidade de vida na doença de Sjögren

Questionário de qualidade de vida para pacientes com doença de Sjögren

Essas perguntas são relacionadas às suas queixas nas últimas 4 semanas.

1.Qual foi a intensidade da sua dor?

0	1	2	3	4	5	6	7	8	9	10
Sem dor										Dor insuportável

2.Eu tive dor nas juntas:
( ) Não ( ) Sim

3.Eu tive dores migratórias recorrentes no meu corpo: (dores que aparecem e desaparecem em diferentes partes do corpo)
( ) Não ( ) Sim

Eu tive problemas digestivos
4.Constipação intestinal: ( ) Não ( ) Sim
5.Dor abdominal/estomacal: ( ) Não ( ) Sim

6.Eu tive problemas no sono
( ) Não ( ) Sim

7.Você tem sentido sua boca seca?
( ) Não ( ) Sim
Se sim: Você teve alguma dessas queixas? (Marque todas que se aplicam)
( ) Sensação de queimação na boca
( ) Dificuldade para falar
( ) Problemas dentários
( ) Alterações no paladar
( ) Dificuldade em comer alimentos secos
( ) Necessidade de beber líquidos durante a noite

8.Você tem sentido seus olhos secos?
( ) Não ( ) Sim
Se sim: Você teve alguma dessas queixas? (Marque todas que se aplicam
( ) Inflamações frequentes (olhos avermelhados)
( ) Dor
( ) Sensação de areia
( ) Excesso de secreção (remela)
( ) Piora da visão
( ) Piora da visão
( ) As atividades diárias como dirigir, ler ou assistir televisão estão limitadas ou impossibilitadas

9.Você tem sentindo a sua pele seca?
( ) Não ( ) Sim
Se sim: Você teve alguma dessas queixas? (Marque todas que se aplicam)
( ) Vermelhidão da pele
( ) Pele esticada

10.Você tem sentindo o seu nariz seco?
( ) Não ( ) Sim
Se sim: Você teve alguma dessas queixas? (Marque todas que se aplicam)
( ) Mudança no olfato (dificuldade de sentir o cheiro)
( ) Sangramento nasal

A próxima pergunta é somente para as mulheres

11.Você tem sentindo a sua vagina seca?

( ) Não ( ) Sim

Se sim: Você teve alguma dessas queixas? (Marque todas que se aplicam)

( ) Algum tipo de dor vaginal

( ) Coceira

( ) Dor na relação sexual

Indique abaixo quais dessas situações se aplicam a você nas últimas 4 semanas:

	Nunca	Raramente	Às vezes	Frequentemente	Sempre
12.Eu tenho sentido que sou a única pessoa com essas queixas.					
13.Eu tenho sentido que minhas queixas não são levadas a sério					
14.Eu tenho sentido que minhas queixas são demais pra mim					
15.Eu tenho sentido que minha família e meus amigos me entendem.					
16. Estou cansado demais para cumprir obrigações com minha família e amigos					
17.Sou retraído, fechado ou isolado.					
18.Estou preocupado (a) com efeitos colaterais dos medicamentos.					
19.Preocupo-me com a progressão da minha doença					
20.Sinto-me bem com o meu corpo					
21. Não consigo lidar com a minha vida diária tão bem como fazia antes de adoecer					
22.Eu me canso facilmente					
23.Atividades rotineiras como dirigir, trabalhar, tarefas domésticas e esportes são um desafio					
24.Tratamentos como colírios, cremes e fisioterapia são uma sobrecarga financeira					
25.A doença tem reduzindo minha qualidade de vida					

Obrigado por responder este questionário!
